# Functions of Small Organic Compounds that Mimic the HNK-1 Glycan

**DOI:** 10.3390/ijms21197018

**Published:** 2020-09-24

**Authors:** Minjuan Wang, Thomas Theis, Maciej Kabat, Gabriele Loers, Lynn A. Agre, Melitta Schachner

**Affiliations:** 1 Keck Center for Collaborative Neuroscience and Department of Cell Biology and Neuroscience, Rutgers University, Piscataway, NJ 08554, USA; minjuan1985@gmail.com (M.W.); Theis@dls.rutgers.edu (T.T.); kabat95@msn.com (M.K.); 2 Zentrum für Molekulare Neurobiologie Hamburg, Universitätsklinikum Hamburg-Eppendorf, 20251 Hamburg, Germany; gabriele.loers@zmnh.uni-hamburg.de; 3 Rutgers School of Arts and Sciences, Department of Statistics and Rutgers Business School, Rutgers University, Piscataway, NJ 08854, USA; agre@business.rutgers.edu

**Keywords:** HNK-1, mimetic, neuritogenesis, neuronal survival, small organic compounds

## Abstract

Because of the importance of the HNK-1 carbohydrate for preferential motor reinnervation after injury of the femoral nerve in mammals, we screened NIH Clinical Collection 1 and 2 Libraries and a Natural Product library comprising small organic compounds for identification of pharmacologically useful reagents. The reason for this attempt was to obviate the difficult chemical synthesis of the HNK-1 carbohydrate and its isolation from natural sources, with the hope to render such compounds clinically useful. We identified six compounds that enhanced neurite outgrowth from cultured spinal motor neurons at nM concentrations and increased their neurite diameter, but not their neurite branch points. Axons of dorsal root ganglion neurons did not respond to these compounds, a feature that is in agreement with their biological role after injury. We refer to the positive functions of some of these compounds in animal models of injury and delineate the intracellular signaling responses elicited by application of compounds to cultured murine central nervous system neurons. Altogether, these results point to the potential of the HNK-1 carbohydrate mimetics in clinically-oriented settings.

## 1. Introduction

Owing to the altogether favorable nature of the mammalian peripheral nervous system to allow regeneration after injury, the question arises how specificity for motor versus sensory reinnervation is achieved. The femoral nerve of the mouse has proven to be accessible for investigation of this topic, since it bifurcates into a motor (quadriceps) and a sensory (cutaneous) branch after exiting from the spinal cord. After injury of the vertebrate femoral nerve before its bifurcation, motor axons specifically regrow into the motor branch, largely ignoring the sensory branch [1,2,3]. In an attempt to find a molecular basis for this astounding specificity in reinnervation, the HNK-1 carbohydrate (hereafter abbreviated HNK1) was found to allow preferential motor reinnervation, a term coined by the pioneering work of Brushart et al. [4,5].

The HNK1 glycan was discovered via a monoclonal antibody obtained by immunization with a human T cell line and found to be predominantly expressed by natural killer cells, hence the name [6]. Its epitope consists of a sulfated or non-sulfated glucuronic acid attached to *N*-acetyllactosamine on glycolipids [7,8] and neural cell adhesion molecules, including the immunoglobulin superfamily molecules neural cell adhesion molecule (NCAM), L1, myelin-associated glycoprotein (MAG), and peripheral nervous system myelin glycoprotein P0 [9,10,11]. Interestingly, not all protein backbones of each molecule express the covalently attached HNK1 glycan but carry it differently at different stages during development and in different parts of the nervous system [12]. Interest in the HNK1 glycan also pertains to its receptors, laminin [13,14], *N*-cadherin [15,16], and high mobility group box 1 (HMGB1, also called amphoterin) [17,18].

As a general strategy, novel compounds with translational issues have first been tested in vitro. This approach was also taken for HNK1. It was substrate-coated in the form of an HNK1 glycolipid and assayed for its ability to elicit neurite outgrowth of mouse spinal motor neurons and dorsal root ganglion neurons. Motor neurons extended longer neurites on this glycolipid than on substrate-coated ganglioside, used as negative control [19]. Similarly-tested dorsal root ganglion neurons did not respond to the HNK1 substrate, as would be expected for preferential motor reinnervation. An 8mer HNK1 mimetic peptide that was proven to be an HNK1 mimetic by competitive enzyme-linked immunosorbent assay (ELISA) using the native HNK1 glycan, thereby validating it firmly as the structural equivalent of the glycan. This mimetic peptide, like the glycan, when used as a substrate coat, also generated longer neurites from motor neurons than the control substrate consisting of a scrambled peptide and did not enhance neurites from dorsal root ganglion neurons [19,20]. These in vitro studies were supported by findings in vivo: the HNK1 mimetic peptide [20] was able to direct preferential motor reinnervation, when applied to the femoral nerve in a conduit—a cuff containing the mimetic together with a supporting scaffold peptide—before bifurcation [21]. Preferential motor reinnervation was shown in mice and in non-human primates, displaying specific motor recovery from femoral nerve injury, with axons regrowing into the motor branch in preference to the sensory branch as verified by behavioral and histological tracing studies [21,22]. It is worth emphasizing that the HNK1 mimetic peptide was used in these in vivo studies, since the HNK1 glycan has been and still is very difficult to isolate from natural sources and complicated to chemically synthesize, thereby not being feasible for in vivo applications and for clinical practice [23,24]. Although less labile regarding degradation in comparison to the glycan, peptides also have the disadvantage to be more easily degraded, and the costs for peptide synthesis and purification can also be substantial. With the aim to provide a novel approach to clinical application, HNK1 mimicking small organic compounds were screened for with commercially available libraries in the hope that they would be successful in promoting neurite outgrowth from motor neurons, but not sensory neurons.

Here we describe the identification of six HNK1 mimicking small organic compounds and show that they resulted in enhanced axonal outgrowth from cultured motor neurons versus sensory neurons. These results raise the hope that such mimetic compounds could be successfully used in clinical situations, where preferential motor reinnervation is the desired endpoint.

## 2. Results

### 2.1. Competitive ELISA Screening

**Figure 1:**
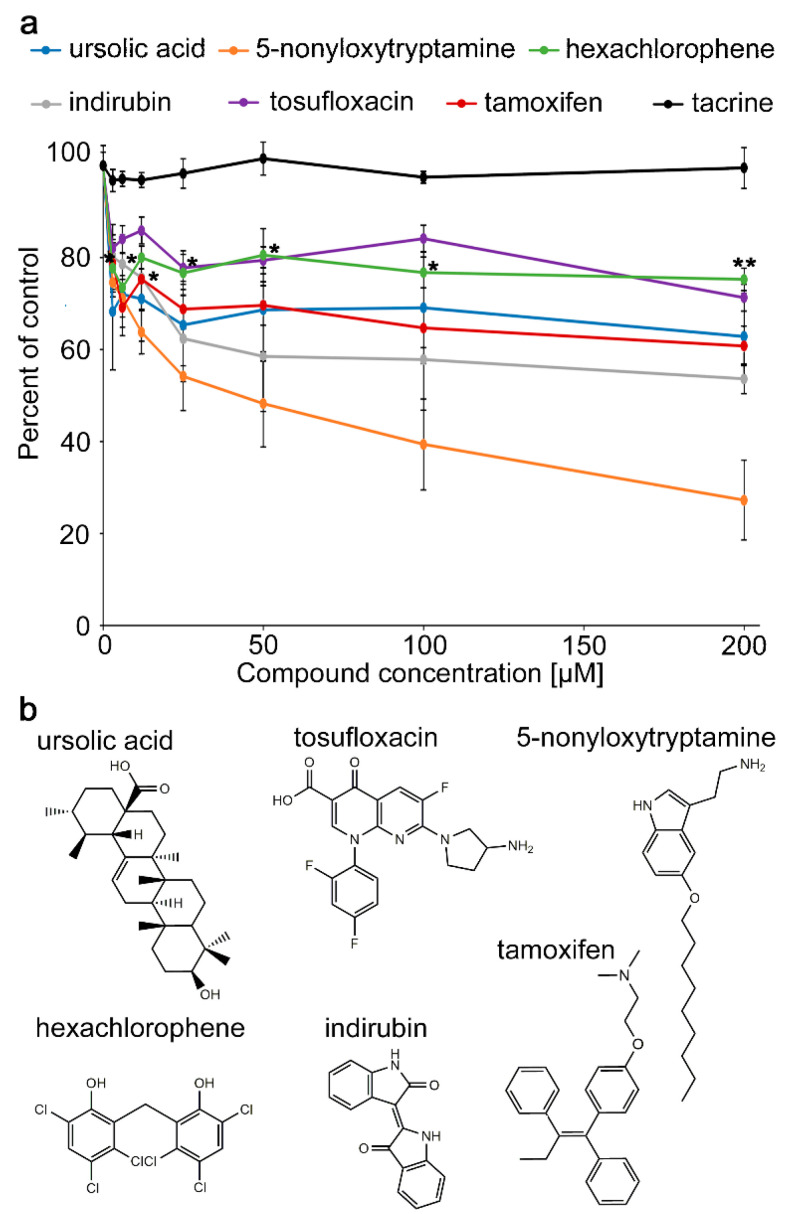
Small organic compounds inhibit the binding of the HNK1-specific antibody to the HNK1 mimetic peptide in a concentration-dependent manner. (**a**) HNK1-specific antibody was substrate-coated on an enzyme-linked immunosorbent assay (ELISA) plate and preincubated with different concentrations (1–200 μM) of compounds from libraries or the negative control compound tacrine. Biotinylated HNK1 mimetic peptide was then added and detected by horseradish peroxidase (HRP)-coupled streptavidin followed by development with o-phenylenediamine dihydrochloride (OPD) substrate to produce a colored product that was quantified in the ELISA reader. The signal with this peptide without compounds was set to 100%. The graph shows average relative inhibition of the signal (duplicate wells carried out in three independent experiments ± SEM). All data points below the asterisks are different in inhibition when compared to the negative control compound tacrine (one-way ANOVA, F(31/64) = 4.122, *p* < 0.0001; Fisher’s PLSD test, * *p* < 0.05, ** *p* < 0.01). (**b**) Structure of the HNK1 mimetic compounds.

Screening of the NIH Clinical Collection 1 and 2 Libraries and a Natural Product library using monoclonal HNK1 antibody (0.5 µg/mL; 100 µL/well) was carried out on substrate-coated mouse brain homogenate (1 mg/mL in PBS). Compounds that inhibited at least 50% of binding of the HNK1-specific monoclonal antibody to the brain homogenate were considered as hits. These potential HNK1 mimicking compounds were thereafter validated with substrate-coated HNK1 antibody (1 µg/mL; 50 µL/well), incubated with each compound, and probed for binding using a biotinylated HNK1 peptide (1 µg/mL in PBS) that was targeted by horseradish peroxidase (HRP)-coupled streptavidin (1:1000 in PBS) (Figure 1). The HNK1 peptide mimetic has been validated in several studies to be structurally and functionally equivalent to the native HNK1 glycan: it was obtained by screening a phage peptide display library with a commercial monoclonal HNK1 antibody and the monoclonal L1 antibody [9], which was thereafter termed HNK1 antibody because their similarity in epitope specific [25]. The HNK1 mimicking peptide was obtained by screening a phage display library using the HNK1-specific antibody by ELISA and its structural identity of the peptide with the glycan was confirmed by competition ELISA: the HNK1 glycolipid blocked the binding of the HNK1 antibody, and vice versa, the peptide blocked the binding of the HNK1 antibody to the HNK1 glycolipid. The HNK1 peptide was synthesized in biotinylated form, and the reverse or random HNK1 peptide served as negative controls. Substrate-coated HNK1 peptide was also used in a competition ELISA, where chemically-generated HNK1 glycan was used to reduce the binding of the monoclonal HNK1 antibody to the peptide in a dose-dependent manner, thereby providing evidence for the structural equivalence of glycan and peptide [20,26]. The functional equivalence of HNK1 mimetic peptide was confirmed in cultures of mouse motoneurons, which increased neurites versus dorsal root ganglion neurons, which were not affected by the HNK1 peptide [19]. Equivalence of the peptide was also shown in vivo with mice and a non-human primate which regained locomotor functions and preferential reinnervation of the quadriceps branch of the femoral nerve as shown by retrograde tracing [5,19,22]. The validated HNK1 mimetic peptide had to be used in the present study because the native HNK1 glycan was no more available.

The following six compounds were validated since they competed more than the negative control compound tacrine (at 200 µM: 99.5% from vehicle control, SEM ± 4.4%): ursolic acid (at 200 µM: 62.8% from vehicle control, SEM + 9.4%) and indirubin (at 200 µM: 53.6% from vehicle control, SEM + 3%) from the Natural Product library; tosufloxacin (at 200 µM: 71.3% from vehicle control, SEM + 6.4%) and 5-nonyloxytryptamine (at 200 µM: 27.2% from vehicle control, SEM + 8.7%) from the NIH Clinical Collection 1 library; and hexachlorophene (at 200 µM: 75.2% from vehicle control, SEM + 2%) and tamoxifen (at 200 µM: 60.8% from vehicle control, SEM + 7%) from the NIH Clinical Collection 2 library (Figure 1). These compounds display different structures, but have in common hydrophobic cores, which could be considered counterintuitive, but such hydrophobic cores have become accepted as features of the overall hydrophilic carbohydrates. That these compounds are structurally dissimilar is noteworthy and may be explained by the possibility that the binding site of the HNK1 antibody is larger in dimension than the size of the mimetics and that binding of a mimetic to one binding site in the pocket of the antibody can inhibit its binding to the antigen. The reason why the hit compounds show an inhibition of antibody binding to the substrate of more than 50% in the initial screen and only one compound inhibited the antibody binding to the substrate by more than 50% in validation can be explained by the fact that we used brain homogenate as substrate for the initial screen and HNK1 mimetic peptide for the validation. The HNK1 epitope in the brain homogenate is distributed on the substrate less densely than the substrate-coated HNK1 mimetic peptide. Thus, a compound was considered validated if it inhibited significantly more the binding to the peptide than negative control, the L1 agonist tacrine.

### 2.2. Neurotoxicity

Before testing whether these compounds would affect neurite outgrowth, it was deemed necessary to determine whether they would be toxic. Since the yield of motor neurons and dorsal root ganglion neurons is too low for Alamar blue assays, we used cultures of cerebellar granule cells to estimate potential neurotoxicity of these compounds (Figure S1). Neurons were treated for 24 h with each of the six compounds at concentrations of 0.001 to 1000 µM. At 1000 µM, all compounds except indirubin reduced cell viability. For 5-nonyloxytryptamine, viability of cells was reduced at 10, 100, and 1000 µM concentrations, but not at lower concentrations. At the tested concentrations of 1 µM and below, none of the other compounds was toxic to the cells.

### 2.3. Neurite Outgrowth

**Figure 2:**
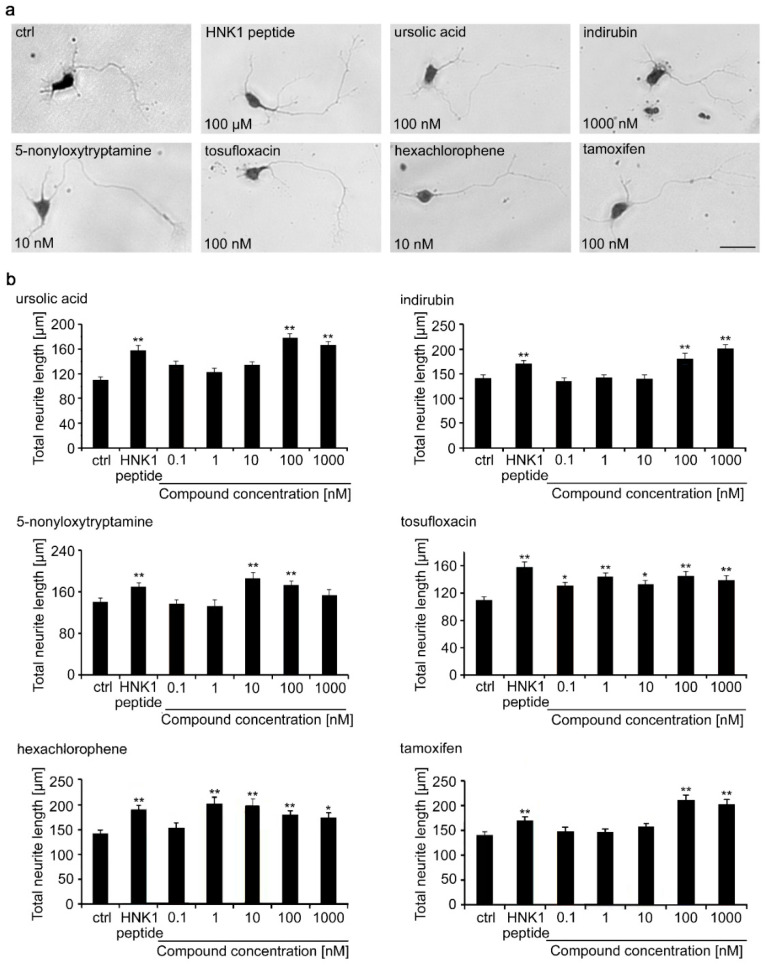
Neurite length of motor neurons treated with different concentrations of HNK1 mimetics as measured by ImageJ analysis. Cultures of motor neurons were treated for 24 h with different concentrations of mimetics and assayed by manual ImageJ analysis. The HNK1 mimetic peptide (100 µM) served as positive control and 0.2% dimethyl sulfoxide (DMSO) was the vehicle control (ctrl). Neurons were fixed and stained with toluidine and methylene blue. (**a**) Representative images show brightfield images of motor neurons that were used to manually measure neurite lengths. Scale bar, 50 µm. (**b**) Bar diagrams show the average total neurite length per neuron (*n* = 300 neurons from three independent experiments + SEM). Asterisks indicate differences in neurite length between HNK1 mimetics and the vehicle control (one-way ANOVA; Fisher’s PLSD test, * *p* < 0.05, ** *p* < 0.01).

Neurite lengths were then determined for motor neurons maintained for 24 h after seeding in the presence of compounds in the range of concentrations between 0.1 and 1000 nM (Figure 2). Compared to the negative vehicle control, the HNK1 mimetic peptide increased neurite outgrowth by approximately 50%. For each of the 6 tested compounds, neurite lengths were slightly, although not statistically significantly longer than on the HNK1 peptide mimetic, most prominently between 100 and 1000 nM for ursolic acid, indirubin, and 5-nonyloxytryptamine. Tosufloxacin showed increased neurite length between 0.1 and 1000 nM. Hexachlorophene led to increased neurite length between 1 and 1000 nM, when compared to the negative control. The HNK1 peptide was used at a 100 µM concentration and the compounds were similarly effective at higher nM concentrations except for tosufloxacin, which was active at a concentration as low as 0.1 nM. It is noteworthy that the small compounds mimicking HNK1 trigger functions at nM concentrations, whereas the HNK1 mimetic peptide triggers neurite outgrowth at µM concentrations. A similar effect was observed with LewisX and polysialic acid mimetics [27,28]. A potential explanation could be that smaller molecules are more flexible in binding into the receptor-binding pocket compared to a larger peptide. In contrast, a peptide might bind with several binding sites into the receptor pocket compared to small organic compounds, which might occupy only one binding site in the receptor pocket. Thus, a higher concentration of small organic compounds is needed to inhibit the binding of a peptide to a receptor. Of note, we observed differences in neurite lengths in different experiments. Preparation of neurons, and in particular motor neurons, has been observed to be variable, often depending on the yearly season and even time of the day [29]. Therefore, standard controls have to be included in each experiment and used to assure the robustness of results. In this study we used the HNK1 peptide as a positive control and the vehicle solution as negative control. All experiments were carried out three times and on different days.

### 2.4. Neurite Diameter

**Figure 3:**
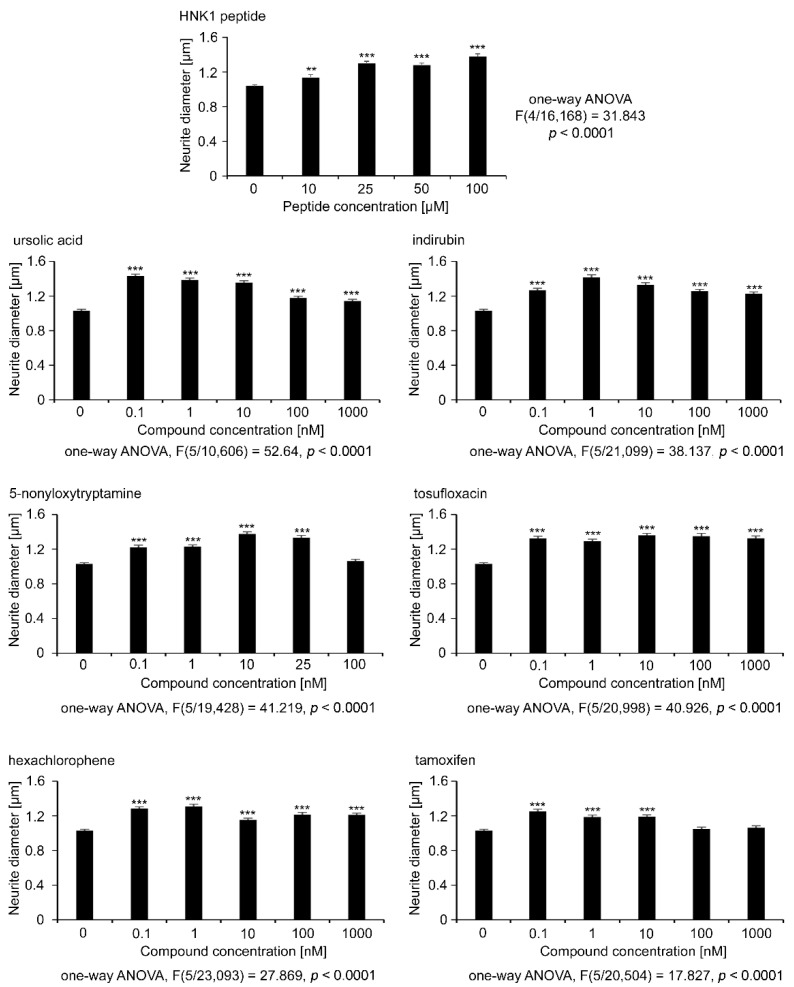
Neurite diameter of motor neurons treated with different concentrations of HNK1 mimetics as measured with automated InCell analysis. Cultures of motor neurons were treated for 24 h with different concentrations of HNK1 mimetics or as positive control with HNK1 mimetic peptide. DMSO (0.2%) served as vehicle control (0). The diameter of β-tubulin class III immunostained neurites was measured with the InCell Analyzer. Bar diagrams show the average neurite diameter per neuron. (*n* ≥ 2000 neurons from three independent experiments + SEM). Asterisks show differences in neurite diameter between HNK1 mimetics and the vehicle control (one-way ANOVA; Fisher’s PLSD test, ** *p* < 0.01, *** *p* < 0.001).

Measurements of neurite diameter were carried out by using the automated InCell microscope (GE Healthcare Life Sciences) and the InCell analysis software setting, which automatically calculate neurite diameter under the premise that neurite vitality is seen by its sturdy process (Figure 3). The average neurite diameter for the vehicle control was 1.03 µm. The HNK1 mimetic peptide increased the neurite diameter in a concentration-dependent manner, starting at 25 µM up to 100 µM with a maximum diameter of 1.37 µm (Figure 3). Interestingly, all mimetic compounds increased the neurite diameter of motor neurons in the broad concentration range of 0.1 nM to 1000 nM, except for 5-nonyloxytryptamine, which showed a similar neurite diameter as the control at 100 nM, and tamoxifen, which had similar values at 100 and 1000 nM concentrations, possibly reflecting a lower cell vitality, although this was not evident at this concentration for the capacity for neurite outgrowth, which was highest for all concentrations tested for this compound. The maximal neurite diameter of approximately 1.4 µm was found with treatments of ursolic acid at 0.1 nM, indirubin at 1 nM, and 5-nonyloxytryptamine at 10 nM.

### 2.5. Branch Points

**Figure 4:**
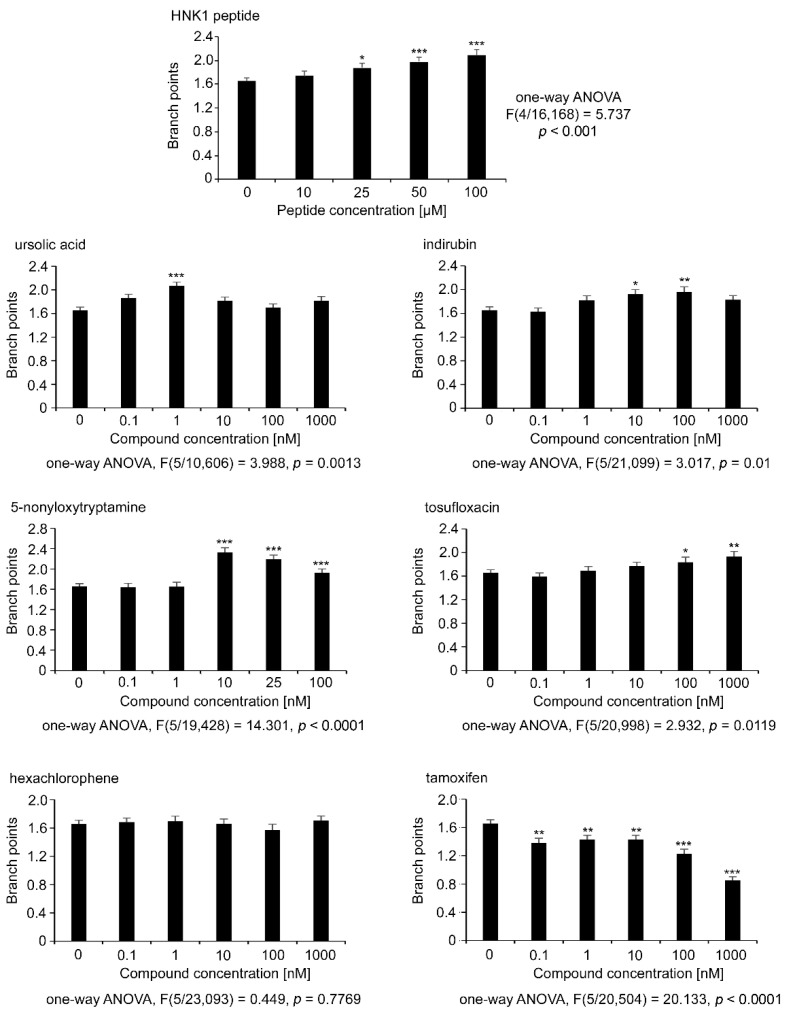
Neurite branch points of motor neurons treated with different concentrations of HNK1 mimetics as measured with automated InCell analysis. Cultures of motor neurons were treated for 24 h with different concentrations of HNK1 mimetics or as positive control with HNK1 mimetic peptide. DMSO (0.2%) served as vehicle control (0). Neurite branch points of β-tubulin class III immunostained neurons were counted by the InCell Analyzer. Bar diagrams show the average number of branch points per neurite (*n* ≥ 2000 neurons from three independent experiments +SEM). Asterisks show differences in branch points between HNK1 mimetics and the vehicle control (one-way ANOVA; Fisher’s PLSD test, * *p* < 0.05, ** *p* < 0.01, *** *p* < 0.001).

Since motor neurons are capable of producing axon collaterals and since these could be biologically relevant in regeneration after injury [30], neurites from motor neurons were tested for this parameter (Figure 4). The automated InCell microscope (GE Healthcare Life Sciences) and the InCell analysis software setting were used to automatically measure the branch points. The HNK1 mimetic peptide increased concentration-dependently the number of branch points per neurite up to 2 (Figure 4). Except for hexachlorophene, compounds showed differences to the control for a branch point that was set to 1.6 as determined for the vehicle control. For ursolic acid a branch point of 2.0 was seen at 1 nM concentration. For indirubin numbers of branch points were slightly less than 2 at 10 nM and 100 nM concentrations. Tamoxifen application resulted in lower branch point numbers than the control at 1.6 for all concentrations.

### 2.6. Dorsal Root Ganglion Neurons

**Figure 5:**
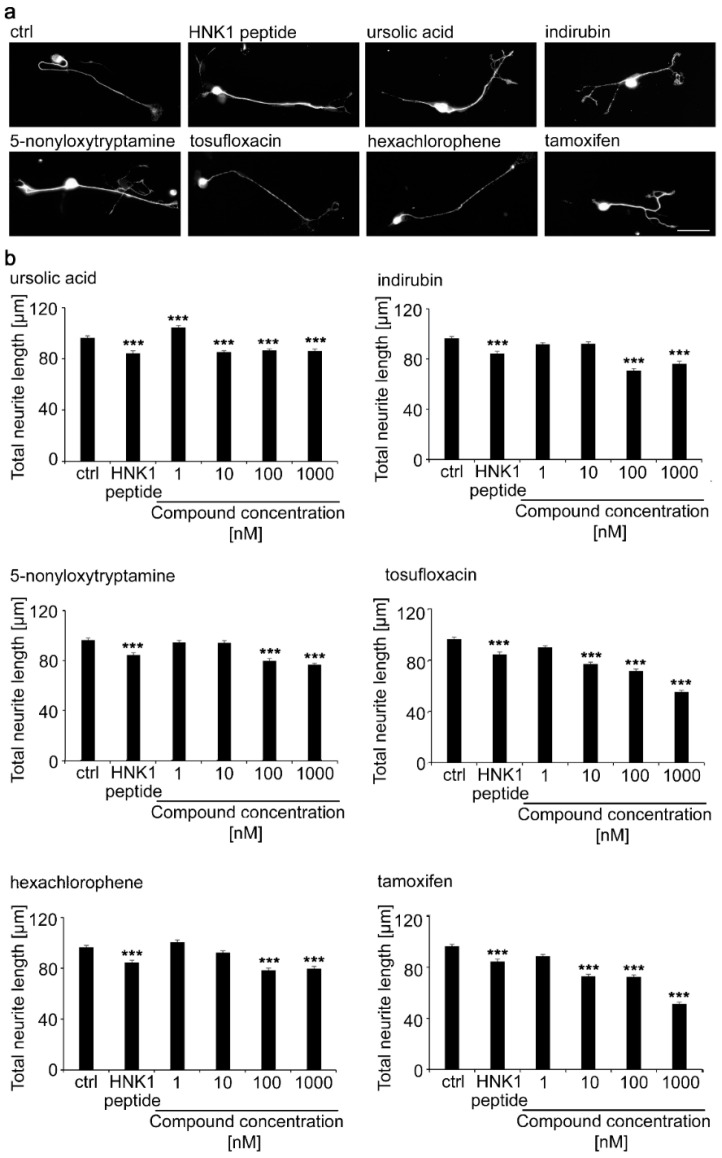
Neurite length of dorsal root ganglion neurons treated with different concentrations of HNK1 mimetics. Cultures were treated with different concentrations of HNK1 mimetics for 24 h. The HNK1 mimetic peptide (100 µM) was used as positive control and 0.2% DMSO served as vehicle control (ctrl). (**a**) Representative images show neurons that were fixed and stained for β-tubulin class III. Neurite length was manually measured by ImageJ. Scale bar, 50 µm. (**b**) Bar diagrams show the average total neurite length per neuron (*n* = 300 neurons from three independent experiments + SEM). Asterisks indicate decreased neurite length with HNK1 mimetics versus vehicle control (one-way ANOVA, *p* < 0.0001; Fisher’s PLSD test, *** *p* < 0.001).

Since in previous studies the neurites of dorsal root ganglion neurons did not respond to the HNK1 carbohydrate [19] and since its HNK1 mimetic peptide did not increase neurite lengths over the substrate control [20], we tested the six HNK1 compounds also with these cells (Figure 5). Except for ursolic acid, all compounds in the range of nM concentrations showed a slightly, but statistically significantly reduced neurite outgrowth. Ursolic acid led to a slightly increased neurite outgrowth at 1 nM. Additionally, the HNK1 peptide reduced neurite outgrowth. These observations may be interesting in interpreting the lack of regrowth of severed dorsal root ganglion axons into the motor branch, which could be reflected by this biologically meaningful reduction of axonal growth in response to the HNK1 glycan.

## 3. Discussion

We have successfully screened for small organic compounds that mimic the structure of the HNK1 carbohydrate using the well-established competitive ELISA. The competitive ELISA performed in the present study used the HNK1 mimetic peptide for screening because it had been validated to be the structural and functional equivalent of the native HNK1 glycan in several studies, thus obviating the demanding chemical synthesis and cumbersome isolation from femoral nerves in the high amounts necessary for large scale screening. HNK1 small organic compounds are interesting in view of the needs to generate modality-specific reinnervation in the peripheral nervous system. It is noteworthy in this context that in the vertebrate peripheral nervous system this epitope is expressed exclusively by the myelin-associated glycoprotein MAG [31], which localizes at the cell surface of the outer myelin loop. This is the site chosen by regrowing axons after injury and is thus readily accessible to these without the need for myelin to degenerate in order to expose functional epitopes. Other adhesion molecules of the immunoglobulin superfamily that can differentially express this glycan do not express it in the motor branch of the femoral nerve. Presumably, a similar selective feature is displayed in the human cerebellum, where the HNK1 epitope is present in stripes of cerebellar Purkinje cells, leading to precise targeting of afferents [32]. This precision may reflect the precision needed in the peripheral nervous system [4,19,33,34]. It is noteworthy in this context that HMGB1 interacts with the receptor for advanced glycation end products (RAGE) via yet undefined carbohydrates. RAGE-associated HNK1 might be one potential candidate of these unknown carbohydrates, which could form an association that contributes to not only neurite outgrowth [35,36], but also to metabolic homeostasis, which is problematic in diabetes.

The identification and characterization of HNK1 mimetic compounds thus appears to be of wider interest than only for the peripheral nervous system. For a translational approach and a clinical setting, it may be worth mentioning that all small compounds act at nanomolar to micromolar concentrations in vitro. Although one can expect 10 to 100 times higher concentrations to show such effects in vivo, these concentrations could be acceptable pharmacologically and economically. Importantly, within the range of these doses, ursolic acid and 5-nonyloxytryptamine have improved functional recovery in injured mice [37,38,39], with the ursolic acid used by Sahu et al. [36] to show its pro-active functions in spinal cord regeneration, being derived from the screen described in the present study. Importantly also, several HNK1 mimetics have been FDA approved for other types of indications.

Here, as also in other publications [27,28], we observed that some glycomimetics have a positive functional effect at lower concentrations, which is attenuated or even abolished at higher concentrations. Low doses of 5-nonyloxytryptamine and tamoxifen increase the neurite diameter, whereas no difference from the vehicle control was observed at higher concentrations. For instance, a similar phenomenon was observed with fibroblast growth factor 2 that enhances cell proliferation, differentiation, and survival at lower concentration, but has the opposite effect with differential signaling, including negative feedback inhibition at higher concentrations [40].

It is important to point out that several HNK1 mimetics have shown other functions than those related to HNK1. Ursolic acid, a pentacyclic triterpene first identified in epicuticular waxes of apples and other plants [41], affects key cell signaling pathways, such as signal transducers and activators of transcription (STATs), nuclear factor (NF)-κB, and tumor necrosis factor-related apoptosis-inducing ligand (TRAIL), and shows anti-bacterial, anti-inflammatory, and anti-tumor features [42,43,44]. In the nervous system, ursolic acid is protective by reducing radiation impairment of neurogenesis, learning, and memory [45]. Furthermore, we found that ursolic acid improves functional recovery after spinal cord injury in mice [38]. Interestingly, ursolic acid is also a mimetic for the terminal trisaccharide LewisX, which plays an essential role in the development and repair of the nervous system, and represents an important post-translational modification of neural cell adhesion molecules such as L1CAM and contributes to enhancing neurite outgrowth and cell viability of cerebellar granule cells [28]. Tosufloxacin, a fluoroquinolone antibiotic, can cause severe thrombocytopenia and nephritis [46]. We had identified ursolic acid as a LewisX mimetic [28] and showed that it triggers signal transduction mechanisms underlying neurite outgrowth and cell survival in a similar manner as tosufloxacin does, with the involvement of protein kinase C, Src, Fyn, and ERK [28].

5-Nonyloxytryptamine, a 5-HT1B serotonin receptor agonist [47], was identified to be a mimetic also for polysialic acid, which is predominantly attached to the neural cell adhesion molecule NCAM and essential not only for development but also for maintenance and repair of nervous system functions in the adult [48,49]. It improves recovery after injury of the central nervous system when injected into the cerebrospinal fluid or applied on collagen-laminin scaffolds [37,39]. It also contributes to synaptic activity and plasticity [49].

Hexachlorophene, a highly lipophilic chlorinated bisphenol, was once widely used as disinfectant in hygienic and dermatological products. In 1972, the FDA restricted the production and distribution of hexachlorophene because of concerns with brain damage in infants exposed to a higher dose (3% or more of the product volume) [50], possibly due to its epigenetic disturbance of gene-regulation in a GABAergic interneuron subpopulation of the hippocampal dentate gyrus [51].

Indirubin, a component of indigo naturalis, which is derived from the leaves and stems of indigo plants, is used in traditional Chinese medicine to treat inflammatory bowel disease [52]. A study of the molecular structure of indirubin indicates that it has a more three-dimensional structure compared to the more planar structure of the indigo molecule which is another component of indigo naturalis [53]. Indirubin is also a potent cyclin-dependent kinase inhibitor which is neuroprotective, attenuates high fat-high fructose induced Aβ-aggregation, and prevents tau-phosphorylation in Alzheimer’s disease animal models [54,55,56,57,58,59,60].

Tamoxifen, a selective estrogen receptor modulator, which is used to treat breast cancer [61], is widely used to induce Cre-recombinase expression in many animal models of human diseases [44]. It is neuroprotective by scavenging reactive oxygen species (ROS), decreasing formation of peroxynitrated proteins and nitric oxide synthase (NOS), inhibiting excitatory amino acid release, and stimulating an anti-inflammatory response [62,63,64,65,66]. In addition, tamoxifen stimulates proliferation and maturation of oligodendrocyte progenitor cells [67]. After chronic cerebral hypoperfusion, tamoxifen improves the recovery of white matter and cognitive functions [68]. These functions of the HNK1 mimetics are not likely to adversely affect cultured motor neurons but need to be considered if HNK1 compounds are applied to patients in need of preferential motor reinnervation or maintenance of myelin integrity in association with axons of motor branches. Altogether, we would like to argue that these functions of the HNK1 mimetics could be taken as a benefit acting in addition to HNK1-specific functions, which would have to be evaluated for each compound in a clinical setting.

## 4. Materials and Methods

### 4.1. Animals

For all experiments, CD-1 IGS mice were used (Charles River, Wilmington, MA, USA; strain code 022). Mice were maintained with ad libitum access to food and water on a 12-h light and 12-h dark cycle in the animal facility of the Division of Life Sciences at the Nelson Biology Laboratories of Rutgers University. Both sexes were used for all experiments. The Institutional Animal Care and Use Committee of Rutgers University approved all experiments (protocol #09-051, 01 May 2017).

### 4.2. Materials

All chemicals were from Sigma-Aldrich (St. Louis, MO, USA) if not indicated otherwise. Media and reagents for cell culture were from Gibco (Gaithersburg, MD, USA). The HNK1 mimetic compounds ursolic acid (CAS 77-52-1; cat #sc-200383), 5-nonyloxytryptamine oxalate (2-[5-(nonyloxy)-1H-indol-3-yl]ethanamine ethanedioate, CAS 157798-13-5; cat #sc-203480), indirubin (isoindogotin, CAS 479-41-4; cat #sc-201531), and hexachlorophene (hexachlorofen, CAS 70-30-4; cat #sc-211587) were from Santa Cruz Biotechnology (Santa Cruz, CA, USA). The HNK1 mimetic compounds tamoxifen (Z)-1-(p-dimethylaminoethoxyphenyl)-1,2-diphenyl-1-butene, trans-2-[4-(1,2-diphenyl-1-butenyl)phenoxy]-*N,N*-dimethylethylamine, CAS 10540-29-1; cat #13258) and tosufloxacin (tosylate) (7-(3-amino-1-pyrrolidinyl)-1-(2,4-difluorophenyl)-6-fluoro-1,4-dihydro-4-oxo-1,8-naphthyridine-3-carboxylic acid 4-methylbenzenesulfonate, CAS 115964-29-9; cat #21427) were from Cayman Chemical (Ann Arbor, MI, USA). L1 mimetic compound tacrine (9-amino-1,2,3,4-tetrahydroacridine hydrochloride, CAS 1684-40-8; cat #sc-200172) was from Santa Cruz Biotechnology (Dallas, TX, USA). The NIH Clinical Collection 1 and 2 Libraries were from Evotec (Hamburg, Germany) and the Natural Product Library was from Selleckchem (Houston, TX, USA). The monoclonal IgM antibody against the HNK1 carbohydrate was from Sigma Aldrich (Cat #C6680, Lot #094M4812). The antibody against class III β-tubulin (cat #A01203-40) was from GenScript (Piscataway, NJ, USA). The secondary donkey anti-mouse IgM antibody coupled to horseradish peroxidase (HRP) (cat #715-035-020) and secondary donkey anti-rabbit IgG antibody coupled to alexa fluor^®^ 488 (cat #711-545-152) were from Jackson ImmunoResearch (West Grove, PA, USA). Streptavidin coupled to HRP (cat #S911) and the AlamarBlue™ Cell Viability Reagent (cat# DAL1025) were from Thermo Fisher Scientific (Waltham, MA, USA). The biotinylated HNK1 peptide (FLHTRLFV) was from GenScript.

### 4.3. Competitive Enzyme-Linked Immunosorbent Assay (ELISA)

To identify small organic compounds that are structural and functional mimetics of the HNK1 carbohydrate, libraries were screened as described [27,28,69]. The HNK1-specific monoclonal antibody used in the present study reacts with the sulfated and non-sulfated sugar chain. To identify the compounds that inhibit the binding of this antibody to the HNK1 peptide, the NIH Clinical Collection 1 and 2 Libraries and the Natural Product Library were screened via competitive ELISA. With this method, our group had identified structural and functional mimetic compounds of the glycans polysialic acid and LewisX [27,28,69,70]. In brief, mouse brain homogenate from 7-day-old wild-type mice solubilized in 1% Triton X-100 and cleared from the insoluble fraction by centrifugation at 1000× *g* for 5 min at 4 °C was substrate-coated in 96-well plates with medium binding surface (Greiner Bio-One, cat #675001) overnight at 4 °C (1 mg/mL in phosphate buffered saline (PBS); 100 µL/well). Subsequently, plates were washed three times with PBS and blocked with 1% bovine serum albumin (BSA) for 1 h at 22 °C. The anti-HNK1 antibody (0.5 µg/mL; 100 µL/well) was pre-incubated for 1 h at 22 °C with either PBS serving as negative control, or PBS containing 1% dimethyl sulfoxide (DMSO) serving as solvent control, or 10 µM of library compounds in 1% DMSO. Subsequently, the antibody/compound mixtures were added to the brain homogenate-coated wells for 1 h at 22 °C, being then treated three times with PBS, incubated with secondary anti-mouse IgM antibody coupled to HRP (1:5000 in PBS), washed again, and incubated for 20–30 min at 22 °C with peroxidase substrate (1 mg/mL o-phenylenediamine dihydrochloride (OPD) dissolved in 50 mM citric acid, 50 mM sodium phosphate, and 0.03% hydrogen peroxide). The reaction was stopped with 2.5 M sulfuric acid and the absorbance was measured at 450 nm with an ELISA reader (EL800; BioTek Instruments, Winooski, VT, USA). A compound was considered a hit if it inhibited at least 50% of binding of the HNK1-specific monoclonal antibody to the brain homogenate.

Since the HNK1 antibody binds also to the HNK1 mimetic peptide (Figure S2), the identified compounds were validated on this substrate by competitive ELISA using the HNK1 mimetic peptide: Antibody was coated in 96-well plates overnight at 4 °C (1 µg/mL; 50 µL/well). Wells were then incubated for 1 h at 22 °C with increasing concentrations (2.5, 5, 10, 25, 50, 100, and 200 µM) of library compounds, with the cell adhesion molecule L1 mimetic compound tacrine [70] serving as negative control. Wells were washed three times with PBS and biotinylated HNK1 mimetic peptide (1 µg/mL in PBS) was added for 1 h at 22 °C. After three washes with PBS, wells were incubated for 1 h at 22 °C with HRP-coupled streptavidin (1:1000 in PBS) and washed again three times with PBS. Finally, peroxidase substrate was added, and the absorbance was determined as described in the previous paragraph. A compound was considered an HNK1 mimetic if it inhibited the HNK1 antibody binding to the HNK1 peptide more than the L1 mimetic compound tacrine.

### 4.4. Cultures of Mouse Primary Neurons

Motor neurons were dissociated from the spinal cord of 13- to 15-day-old mouse embryos as described [21]. In brief, spinal cords were washed once with ice cooled Ham’s F12 and incubated with 0.25% trypsin, 0.1% collagenase in Ham’s F12 for 30 min at 37 °C, thereafter washed two times with ice cooled Ham´s F12, and dissociated in 0.01% DNase I in Ham´s F12 with fire polished glass Pasteur pipettes. The dissociated cells were loaded on a 25% and 50% Optiprep gradient and centrifuged for 20 min at 400× *g* and 4 °C. Motor neurons were collected from the interface between the 50% and 25% Optiprep solution and diluted with Ham’s F12. Subsequently, cells diluted in Ham’s F12 were loaded on top of a 3.5% BSA cushion in Hanks’ Balanced Salt solution (HBSS) without Ca^2+^ and Mg^2+^ and centrifuged for 15 min at 100× *g* and 4 °C. Neurons were seeded (40,000 cells/mL) into 0.01% poly-d-lysine coated 96-well culture plates and maintained in motor neuron medium (Neurobasal medium with 30 nM sodium selenite, 8 ng/mL hydrocortisone, 20 nM progesterone, 29 μg/mL putrescine, 9 μg/mL insulin, 5 μg/mL transferrin, 9 μg/mL BSA, 1% Pen/Strep, and 1x B27) at 37 °C and in 5% CO2.

Dorsal root ganglion neurons were prepared as described [71,72]. In brief, dorsal root ganglia were dissected from 15-day-old mouse embryos, cleaned, and collected in ice-cold HBSS. Ganglia were incubated with 0.5% collagenase in HBSS for 45 min at 37 °C. Half of the collagenase solution was replaced with an equal volume of 0.25% trypsin in HBSS. Ganglia were then incubated in this solution for 30 min at 37 °C, washed two times with dorsal root ganglion medium (Neurobasal A with 2 mM l-glutamine, 0.1% BSA, 1% Pen/Strep, and 2% B27) and dissociated with fire polished glass Pasteur pipettes. Neurons were seeded (100,000 cells/mL) into 0.01% poly-d-lysine-coated 96-well culture plates and maintained in dorsal root ganglion medium.

### 4.5. Neurite Outgrowth

Neurite outgrowth measurements were performed as described [28]. In brief, primary motor neurons and dorsal root ganglion neurons were prepared, respectively, from embryonic and early postnatal mice and seeded (30,000 cells/well) into 0.01% poly-d-lysine 48-well Falcon tissue culture plates (Thermo Fisher Scientific, cat# 0720086). Cells were allowed to settle for 1 h and then incubated with different concentrations of the HNK1 mimetics (0.1, 1, 10, 100 nM, and 1 µM), with 0.2% DMSO as solvent control, or with HNK1 glycomimetic peptide (100 µM). Neurons were maintained for 24 h at 37 °C in 5% CO2. Motor neurons were fixed and stained in two different ways, which depended on how the neurons were analyzed: (1) if the neurite length were manually traced and analyzed with ImageJ software, then the motor neurons were fixed with 2.5% glutaraldehyde for 30 min at 22 °C and stained with 1% toluidine blue and 0.1% methylene blue in 1% Na-tetraborate. Neurites were imaged and quantified using an Axio Observer.A1 microscope (Carl Zeiss) with a 20× objective and AxioVision 4.6 software. The total neurite lengths per cell soma were measured from the edge of the cell body to the end of the processes, taking into account only neurites with a length equal to or greater than the diameter of the cell soma from which they originated and only from those that showed no contact with other neurites or cell bodies. All measurements were performed with ImageJ software. (2) If the neurite diameter and branch points were automatically imaged and quantified using an InCell microscope (GE Healthcare Life Sciences), then the motor neurons were fixed with 4% formaldehyde for 15 min at 22 °C and immunostained for β-tubulin class III. Wells that contained the differently treated motor neurons were imaged, and branch points and neurite diameter of every neurite in these wells were measured with the InCell analyzing software (GEHealthcare Life Sciences). Dorsal root ganglion cells were fixed with 4% formaldehyde for 15 min at 22 °C and immuostained for β-tubulin class III. Neurite length was measured with ImageJ software.

### 4.6. Statistical Analysis

All experiments were performed and analyzed in a blinded manner as described (Theis et al. 2018). Each experiment was performed at least three independent times and the results reflect the averages of all experiments. Statistical comparisons between groups were performed by one-way ANOVA using Fisher’s protected least significant difference (PLSD) test. StatView Version 5.0.1 (SAS Institute Inc., New York, NY, USA) and Microsoft Excel (Redmond, Washington, USA) were used for all calculations (verified by SPSS, Version 25, IBM, 2017). Chemical drawings were prepared with ChemDraw Professional 16.0 Suite (Perkin Elmer, Waltham, MA, USA).

## 5. Conclusions

The present study shows that the complex glycan HNK1 can be reduced in its functions to simpler structures, such as small organic compounds without loss of a link to carbohydrate-based biology as indicated by its expected tropism on motor but not sensory neurons. In the past, we had shown that an HNK1 mimicking peptide can perform HNK1′s functions by demonstrating the structural equivalence of the mimetic with the native glycan. The peptide mimetic now served in the present study as the glycan surrogate by mimicking HNK1 functions in cell cultures. This approach needed to be taken, since the native glycan was no more available. Thus, the HNK1 mimicking peptide served as bona fide intermediate link to the native carbohydrate from natural sources, thereby allowing the validation of the small molecule mimetics as chemical structures that are capable to subserve the native biological functions of the HNK1 glycan. In the future, experiments will have to be carried out to evaluate the influence of HNK1 mimetics on neurite outgrowth and neuronal survival in vitro and in vivo. In vitro, co-cultures of motor neurons with different types of muscle cells will be performed using markers for pre- and postsynaptic molecules. Specific target selection will be measured. In vivo, preferential motor reinnervation will have to be studied, as previously performed with mouse and monkey. Signal transduction measurements underlying neurite outgrowth and neuronal survival should serve as a first step towards the application of mimetics in clinical settings.

## Supplementary Materials

Supplementary materials can be found at https://www.mdpi.com/1422-0067/21/19/7018/s1.

## Abbreviations

BSA	bovine serum albumin
DMSO	dimethyl sulfoxide
ELISA	enzyme-linked immunosorbent assay
HBSS	Hanks’ balanced salt solution
HMGB	high mobility group box 1
HNK1	HNK-1
HRP	horseradish peroxidase
MAG	myelin-associated glycoprotein
NCAM	neural cell adhesion molecule
NF	nuclear factor
OPD	o-phenylenediamine dihydrochloride
PBS	phosphate buffered saline, pH 7.3
RAGE	receptor of advanced glycation end products
STAT	signal transducer and activator of transcription
TRAIL	tumor necrosis factor related apoptosis-inducing ligand
