# Supplementary material for: Functions of Small Organic Compounds that Mimic the HNK-1 Glycan
Source: Int J Mol Sci. 2020 Sep 24;21(19):7018. doi: 10.3390/ijms21197018 (PMC7582369; doi:10.3390/ijms21197018)
Supplement: Supplementary file 1 [file ijms-21-07018-s001.pdf]

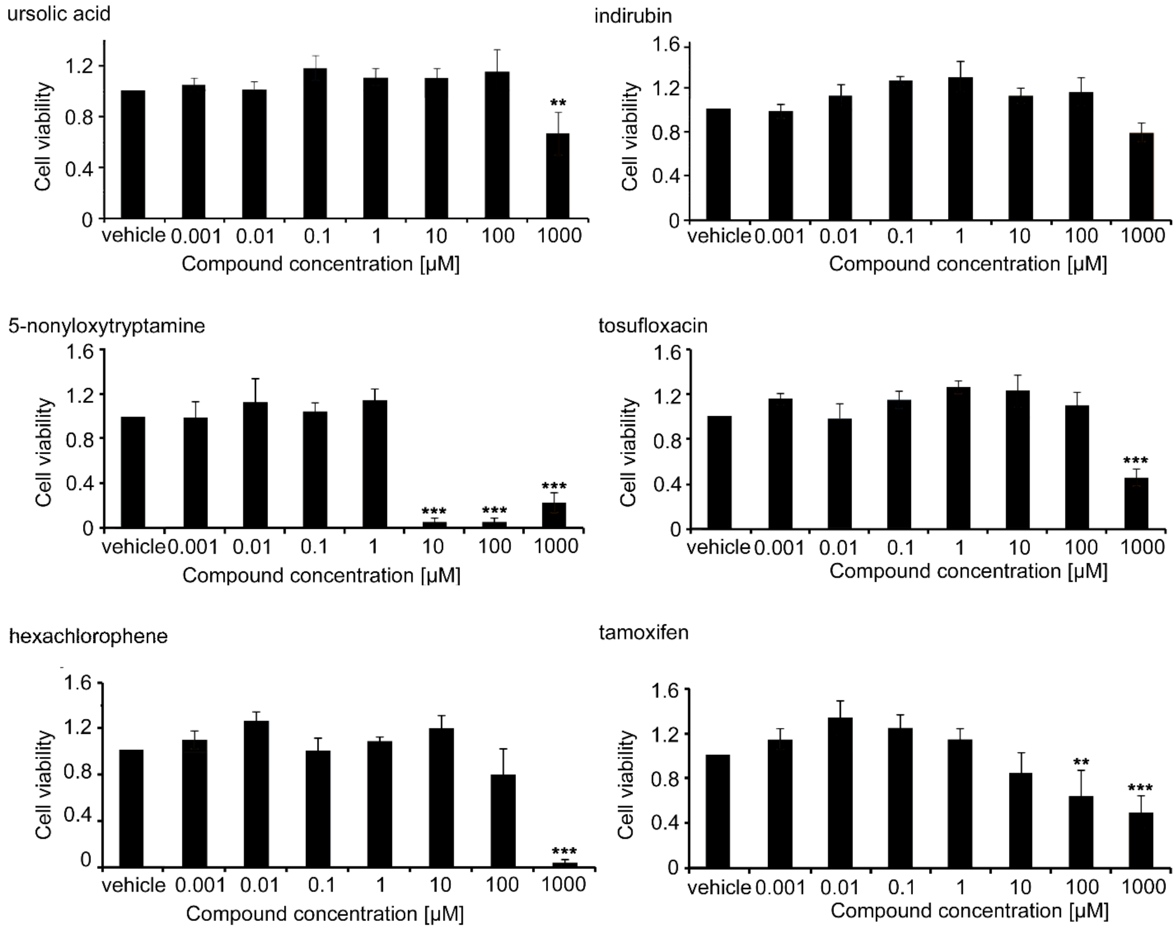

Figure S1: Neuronal survival at different concentrations of HNK1 mimetics. Cerebellar granule cells were prepared (Theis et al. 2018) and seeded on 0.01% poly-D-lysine-coated 96-well Falcon tissue culture plates. Neurons were treated for 24 h with different concentrations (0.001, 0.01, 0.1, 1, 10 100, 1000 µM) of each mimetic. The non-toxic concentration range of the HNK1 mimetics was estimated using the AlamarBlue™ Cell Viability Reagent according to the manufacturer's instructions. Bar diagrams show viability of cells treated with HNK1 mimetics (n=6 wells from three independent experiments + SEM) in comparison to vehicle control (0.2% DMSO). Asterisks show differences in cell viability between HNK1 mimetics and vehicle control (one-way ANOVA, $F(42/256) = 10.535$, $p < 0.0001$; Fisher's PLSD test, $^{**}p < 0.01$, $^{***}p < 0.001$).

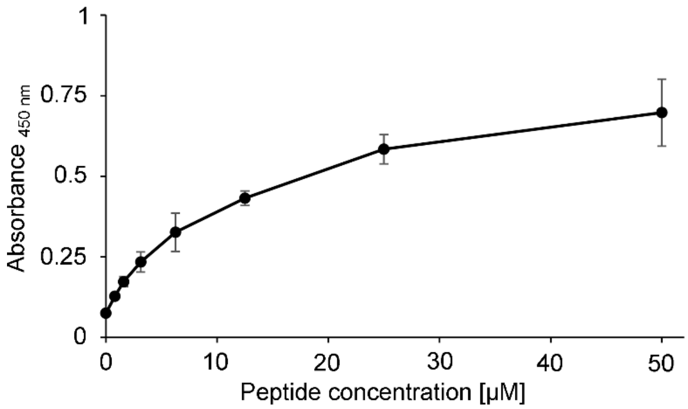

Figure S2: Concentration-dependent binding of the HNK1 mimetic peptide to the HNK1-specific antibody. Monoclonal HNK1-specific antibody was tested by ELISA in a concentration-dependent manner for its capacity to bind to the HNK1-specific mimetic peptide. HNK1-specific antibody was substrate-coated in 96-well plates overnight at 4°C (1 µg/ml; 50 µl/well). Wells were then incubated for 1 h at 22°C with increasing concentrations (0.8, 1.6, 3, 6, 12.5, 25, and 50 µM) of biotinylated HNK1 mimetic peptide. After three washes with PBS, wells were incubated for 1 h at 22°C with streptavidin coupled to horseradish peroxidase (1:10,000 in PBS) and washed again three times with PBS. Peroxidase substrate (1 mg/ml OPD) was then added to the wells. After 20-30 min, 2.5 M sulfuric acid was added to stop the reaction and absorbance was measured. Graph shows the average absorbance (n=6 wells, from three independent experiments ± SEM).
